# Systemic T Cells Immunosuppression of Glioma Stem Cell-Derived Exosomes Is Mediated by Monocytic Myeloid-Derived Suppressor Cells

**DOI:** 10.1371/journal.pone.0169932

**Published:** 2017-01-20

**Authors:** Rossana Domenis, Daniela Cesselli, Barbara Toffoletto, Evgenia Bourkoula, Federica Caponnetto, Ivana Manini, Antonio Paolo Beltrami, Tamara Ius, Miran Skrap, Carla Di Loreto, Giorgia Gri

**Affiliations:** 1 Department of Medical and Biological Sciences, University of Udine, Udine, Italy; 2 Department of Neurosurgery, University Hospital of Udine, Udine, Italy; Istituto Superiore Di Sanita, ITALY

## Abstract

A major contributing factor to glioma development and progression is its ability to evade the immune system. Nano-meter sized vesicles, exosomes, secreted by glioma-stem cells (GSC) can act as mediators of intercellular communication to promote tumor immune escape. Here, we investigated the immunomodulatory properties of GCS-derived exosomes on different peripheral immune cell populations. Healthy donor peripheral blood mononuclear cells (PBMCs) stimulated with anti-CD3, anti-CD28 and IL-2, were treated with GSC-derived exosomes. Phenotypic characterization, cell proliferation, Th1/Th2 cytokine secretion and intracellular cytokine production were analysed by distinguishing among effector T cells, regulatory T cells and monocytes. In unfractionated PBMCs, GSC-derived exosomes inhibited T cell activation (CD25 and CD69 expression), proliferation and Th1 cytokine production, and did not affect cell viability or regulatory T-cell suppression ability. Furthermore, exosomes were able to enhance proliferation of purified CD4+ T cells. In PBMCs culture, glioma-derived exosomes directly promoted IL-10 and arginase-1 production and downregulation of HLA-DR by unstimulated CD14+ monocytic cells, that displayed an immunophenotype resembling that of monocytic myeloid-derived suppressor cells (Mo-MDSCs). Importantly, the removal of CD14+ monocytic cell fraction from PBMCs restored T-cell proliferation. The same results were observed with exosomes purified from plasma of glioblastoma patients. Our results indicate that glioma-derived exosomes suppress T-cell immune response by acting on monocyte maturation rather than on direct interaction with T cells. Selective targeting of Mo-MDSC to treat glioma should be considered with regard to how immune cells allow the acquirement of effector functions and therefore counteracting tumor progression.

## Introduction

Patients with glioblastoma (GBM) are locally and systemically immunosuppressed [1,2] as lymphocyte counts, mainly CD4+, are reduced and T-cell proliferation, in response to interleukin-2 (IL-2), is impaired [3]. Moreover, it has emerged that circulating immunosuppressive cells, such as CD4+/CD25+/FoxP3+ regulatory T (Treg) cells [4] and myeloid-derived suppressor cells (MDSC) [5], are increased in GBM patient’s blood compared to that of a healthy individual. Surgical removal of the primary tumor can result in the restoration of peripheral T cells response to mitogens *in vitro*, an effect that declines with tumor recurrence [6].

To elucidate the mechanism underneath the glioma-mediated immunosuppression, it has been reported that GBM cells directly secrete multiple immunosuppressive factors such as transforming growth factor-β2 (TGF-β2) and prostaglandin E_2_ (PGE_2_), that suppress lymphocytes proliferation, and IL-6, that shift adaptive immunity to a humoral T helper 2 (Th2) type of response (reviewed in [7,8]). Cultures of GBM cell lines produce a soluble factor(s) that inhibits stimulated T cells proliferation and IL-2 production [9]. Moreover, GBM cell-derived supernatants induce down-regulation of IL-12, MHC class II and CD80/86 costimulatory molecules and concomitant up-regulation of IL-10 in stimulated monocyte isolated from healthy individuals [9]. Some evidence suggests the involvement of glioma cancer stem cells (GSC), a population of undifferentiated cells with the capacity for self-renewal and the generation of a tumor upon intracranial transplantation, that recapitulates the cellular heterogeneity present in the parental glioma [10], in tumor-mediated immunosuppression. It has been reported that GSC have reduced expression of MHC and co-stimulatory molecules but demonstrate high levels of immune-inhibitory molecules [11]. Moreover, GSC supernatants inhibit T cell proliferation and activation, induce T-reg cells, trigger T-cell apoptosis [12] and modulate innate immunity by inducing immunosuppressive macrophages/microglia [13]. These findings suggest that one or more factors present in the supernatants of GSC culture exert immunoregulatory effects at both systemic cellular immunity and primary tumor site.

Exosomes are small (30 to 100 nm) membrane vesicles of endocytic origin, containing a variety of molecules including signal peptides, mRNA, microRNA and lipids, that are released from normal, diseased and neoplastic cells [14]. Exosomes are present in the blood and in other bodily fluids together with other extracellular vesicles (EVs) of different size, intracellular origin and composition, such as micro vesicles (MVs) (100–1000 nm) and apoptotic bodies that are released during cell death (500–2000 nm) [15]. EVs have been found to be released by GBM cells in which they represent a key mechanism of intracellular signalling able to support GBM cell proliferation, invasiveness, and chemo resistance as well as neoangiogenesis stimulation [16,17]. An increasing number of studies indicate that the physio-pathological role of tumor-derived EVs, including exosomes, might be in favour of supporting immune-suppression, as they contribute to the cancer progression by acting on a variety of immune cell types, including effector T cells, naturally occurring T-reg cells and NK cells [18,19].

The immunomodulatory properties of exosomes in neuro-oncology have only recently been investigated. Exosomes and cytokines present in serum of GBM patients promote a Th2 type environment, rich in IL-4, IL-13, and TGF-β, in the peripheral blood [20,21] indicating that tumors exert an immunosuppressive systemic effect beyond the boundaries of the CNS. A previous study revealed that exosomes from murine-derived glioblastoma GL26 cell line promote tumor growth by inhibiting the number and function of cytotoxic CD8^+^ T cells *in vivo* [22]. Moreover, GBM-derived vesicles affect cytokine output and migratory capabilities of mitogen-stimulated healthy peripheral blood mononuclear cells (PBMCs) [23] and skew the differentiation of peripheral blood-derived monocytes to alternatively activated M2 tumor-supportive macrophages [24]. Although not all the aspects related to the exosome-induced tumor progression and tolerance have been understood, exosomes could represent potential glioma biomarkers and specific targets to improve tumor immunotherapy [25].

Here, to shed light on the contribution of GSC-derived exosomes to the inhibition of systemic antigen-specific immune response, we looked at their cellular targets among whole PBMCs.

The effect of GSC-derived exosomes on PBMCs immune response was compared to that on an isolated CD4+ T cell. Proliferation, expression of activation markers and intracellular cytokine profile were examined. We demonstrated that GSC-derived exosomes were able to downregulate T cells response only in the presence of monocytes. This suppression activity was associated with the presence of a population of slightly immature monocytes, namely monocytic (Mo) MDSCs and not with the activation of T-reg cell response.

To confirm these results in a more physiological setting, exosomes derived from plasma of GBM patients were tested, supporting the elucidated mechanism of immune downregulation.

## Materials and Methods

### Patients, GSC isolation and culture, astrocytes and blood sample

The independent ethic committee of the Azienda Ospedaliero-Universitaria of Udine has approved the research (Consent 102/2011/Sper). Written informed consents have been obtained from patients and all clinical investigations have been conducted according to the principles expressed in the Declaration of Helsinki.

GSC were isolated from eight patients affected by a supratentorial glioblastoma arising *de novo*, as previously described [26,27]. Glioma fragments were first disaggregated mechanically with scalpels and then enzymatically dissociated, in a 0.0125% Collagenase type II solution (Worthington) in Joklik modified Eagle’s Medium (Sigma-Aldrich), for 5 minutes at 37°C. Collagenase activity was stopped by the addition of 0.1% bovine serum albumin (Sigma-Aldrich) solution in Joklik modified Eagle’s Medium (Sigma-Aldrich). Cell suspension was centrifuged at 500 g for 10 minutes and filtered through a sieve (BD Falcon) in order to select a population less than 40 μm in diameter. Cells were then seeded at a density of 2x10^4^ cells/cm^2^ onto laminin coated dishes in a growing serum-free medium composed as follows: Neurobasal-A medium (Gibco), 2mM L-glutamine (Sigma-Aldrich), 1X N2 supplement (Gibco), 25μg/ml Insulin, Penicillin-streptomycin, 100μg/ml human apo-trasferrin (Sigma-Aldrich), 1X B-27 supplement (Gibco), 20ng/ml h-FGF-basic (Peprotech), 20ng/ml h-EGF (Peprotech). Cells were cultured in an incubator at 37°C, 5%O2/5%CO2. Half of the medium was replaced with a fresh one every 4 days. Once cells reached 70–80% of confluence, they were detached with TrypLE Express (Invitrogen) and re-plated at a density of 3–4 x 10^3^/cm^2^.

Human astrocytes (ScienCell Research Laboratories) were maintained in Astrocytes Medium (Gibco) supplemented with 10% fetal bovine serum (FBS) (Euroclone) and 1% penicillin/streptomycin (Euroclone). To remove the exosomal fraction present in FBS, serum was always ultracentrifuged for 4 hours at 100,000 g (uFBS).

Blood samples were collected in EDTA from ten GBM patients and 10 healthy donors. To separate plasma, blood was centrifuged at 2,000 g for 15 minutes at 4°C. Plasma was transferred into a clean tube and centrifuged again at 14,000 g for 15 minutes at 4°C before being aliquoted and stored at −80°C until use.

### Isolation of T cell populations and depletion of CD14+ population from PBMCs

For the in vitro experiments, whole blood samples from healthy donors were collected in EDTA-tubes by the Department of Transfusion Medicine (University Hospital of Udine) after a written informed consent was signed.

PBMCs were separated by centrifugation at 700 g for 20 minutes on a Ficoll Hypaque density gradient (Millipore) and resuspended at 1 x 106 cells/ml in RPMI 1640 complete medium supplemented with 10% uFBS, 1% glutamine, 1% Na pyruvate, 1% non-essential aminoacid, 1% penicillin/streptomycin, 1% Hepes and 50μM β-mercapthoethanol (all from Euroclone).

CD4+ T cells were purified from PBMCs by negative selection using a human CD4+ T cell enrichment kit (StemCell Technologies), according to the manufacturer’s instructions. Purity of monocytes was over 95% as proved by staining with anti-CD14 mAb (eBiosciences) and flow cytometry analysis (FACScalibur cytometer, BD, Biosciences).

Treg cells were isolated from PBMC with human CD4+CD25+CD127dim/- Regulatory T cell isolation kit II (Miltenyi Biotec). Purification efficiency was over 95%.

To remove monocytes, PBMC were stained with an anti-CD14 fluorescent antibody (clone 61D3, PE conjugated, eBiosciences) and CD14 negative cells were sorted by FACSAria II cell sorter (BD Biosciences). As a control, unfractioned PBMCs were sorted as well.

### Exosomes isolation from plasma of GBM patients, GSC or astrocytes supernatants

Exosomes were isolated from the plasma using the ExoQuick Exosome Precipitation Solution (System Biosciences) according to the manufacturer’s instructions. Briefly, plasma was incubated with 5 U/ml Thrombin (System Biosciences) for 5 minutes and centrifuged at 10,000 g for 5 minutes. The serum-like supernatant was mixed with ExoQuick solution (63 μl for 250 μl of plasma), incubated at 4°C for 30 minutes and then was centrifuged at 1,500 g for 30 minutes at 4°C. Exo-free supernatant fraction was collected, then concentrated using Amicon Ultra-2 centrifugal filters (Millipore) and used in the indicated experiments. Pelleted exosomes were resuspended in Phosphate-buffered saline (PBS) (equal volume of the initial volume of plasma sample) and stored at −20°C.

For the exosomes’ isolation from GSC or human astrocytes supernatant, 3 x 10^5^ cells were seeded in 7 ml culture medium in 100 mm Petri dishes, supernatant was collected after 48 hours, centrifuged at 3,000 g for 15 minutes at 4°C to remove cells and cell debris, and then filtered through a 0.2 μm filter to remove particles larger than 200 nm. Supernatant and ExoQuick-TC Exosome precipitation solution (System Biosciences) were combined in a 5:1 dilution, incubated overnight at 4°C and exosomes were collected as described above. Pelleted exosomes were resuspended in RPMI 1640 complete medium or RIPA buffer.

To compare results obtained with ExoQuick-purified exosomes with those prepared by ultracentrifugation (UC), the conditioned media from GSC, or plasma from patients and healthy donors, were collected and centrifuged for 15 minutes at 2,000 g before being centrifuged further for 30 minutes at 10,000 g. To isolate the exosomes, the supernatants were ultracentrifuged for 90 minutes at 110,000 g (Optima TL ultracentrifuge, Beckman Coulter), the pellets were washed in PBS and a second ultracentrifugation was performed for 90 minutes at 110,000 g. The exosomes were resuspended in RPMI 1640 complete medium for functional studies.

### Characterization of GSC-derived exosomes

The concentration and size of purified exosomes were measured by nanoparticle tracking analysis (NTA) equipped with a 532 nm laser (Nanosight LM10 system Ltd.) that relates the rate of Brownian motion to particle size. Each sample was filmed on video for 60s with a detection threshold set at 16. The temperature was monitored throughout the measurements. Vesicle size distribution, together with an estimated concentration of NTA profiles, was obtained from the raw data files given by the program itself. Triplicate samples were diluted 1:1000 in sterile-filtered PBS and analysed.

The expression of the exosomal marker TSG101 in GSC cells and GSC-derived exosomes was validated by western blot (WB) analysis. Jurkat T cell lysate and GSC-derived exosome-free supernatant fraction were used as positive and negative controls, respectively. Cells and exosomes were lysed in RIPA buffer supplemented with protease inhibitor (Sigma-Aldrich), quantified by Bradford and 20 μg of proteins were loaded for polyacrylamide gel electrophoresis, then transferred to PVDF membranes for blocking and subsequent probing with primary antibodies against TSG101 (clone 4A10, dilution 1:500, Abcam). Secondary antibodies, conjugated to horseradish peroxidase (dilution 1:1000, Dako), visualized the proteins by way of chemiluminescence (ECL Western blotting substrate, Pierce).

To confirm that we obtained an exosomal subpopulation within EVs [28], exosomes were stained as in reference [26]. Briefly, exosomes were attached to 4 μm aldehyde/sulphate latex beads (Molecular Probes, Invitrogen, Carlsbad, CA), previously coated with an anti-CD63 antibody (BD Biosciences), by mixing 10 μg of exosomes with 2 μl of anti-CD63-coated beads (200000 antibody-coated beads) for 15 minutes at RT. This suspension was diluted to 300 μl of PBS and left for two hours at RT under gentle rotation. The reaction was stopped with 200 mM glycine and the exosomes-bound beads were washed in PBS/Tween 0.05%, blocked with Fc blocker (Miltenyi Biotec) and stained with FITC- or APC-conjugated antibodies specific for CD63 (Santa Cruz Technology Inc), CD81 (Biolegend) and CD9 (AbD Serotec), and the corresponding isotype controls for 30 minutes at RT. The samples were acquired with a FACSCanto II (BD Biosciences) and analysed using Summit software (Dako Cytomation).

### In vitro stimulation of T cells and monocyte and co-culture with exosomes

To determine the immunomodulatory effect of exosomes on PBMCs and CD4+ T cells, 2 x 10^5^ cells resuspended in 200 μl of medium were preincubated for 24 hours with 1.5 x 10^10^ exosomes, corresponding to 0.3 μg of protein, produced by GSC or human astrocytes and then seeded into 96 wells with pre-bound 0.5 μg/ml anti-CD3 (clone OKT3, eBiosciences) and 0.5 μg/ml anti-CD28 (clone CD28.6, eBiosciences). For CD4+ stimulation, 250 U/ml of recombinant human IL-2 (Peprotech) was also adjoined. In some experiments, LPS (200 ng/ml, Sigma-Aldrich) was used to stimulate monocytes in the culture.

In experiments with plasma-derived exosomes, different exosome dilutions (1:2, 1:10, 1:50 and 1:100, with respect to the initial volume of plasma sample) were tested. Negative controls (unstimulated PBMCs) and positive controls (stimulated PBMCs without exosomes) were used in all reported experiments. Exoquick also precipitated the medium used in negative and positive controls.

### Flow cytometry

The phenotype of PBMCs cell subsets was evaluated by multiparameter flow cytometry. Cells were incubated in the dark for 20 min RT with the following panel of anti-human fluorescent labelled antibodies: CD3 (clone HIT3a, APC conjugated, BioLegend), CD4 (clone SK3, APC conjugated, eBiosciences), CD8 (clone SK1, APC conjugated, BD Biosciences), CD14 (clone 61D3, FITC conjugated, eBiosciences), CD25 (clone BC96, Alexa Fluor 488 conjugated, eBiosciences), CD69 (clone FN50, PE conjugated, eBiosciences), CD45 (clone L48, PerCyP conjugated, BD Biosciences), Foxp3 (clone PCH101, PE conjugatated, eBiosciences), IL-1β (clone CRM56, FITC conjugated, eBiosciences), IL-6 (clone MQ2-13A5, FITC conjugated, eBiosciences), IL-10 (clone JES3-9D7, Alexa Fluor 488 conjugated, eBiosciences), HLA-DR (clone L243, APC.Cy7 conjugated, BD Biosciences), CD33 (clone P67.6, PE conjugated, BD Biosciences), CD11b (clone D12, APC conjugated, BD Biosciences), CD14 (clone MɸP9, Brilliant Violet 421 conjugated, BD Biosciences) and Arginase-1 (ARG-1, FITC-conjugated, R&D Systems). Isotype controls were used for each experiment. After incubation, cells were again washed, resuspended in flow buffer and analyzed using FACSCalibur and FACSCanto II cytometers (BD, Biosciences). At least 5 × 10^4^ events were collected, and the data was analyzed using Summit software (Summit Group Software).

### CFSE proliferation, expression of activation markers and viability assay

PBMCs were labelled with 5μM carboxyfluorescein succinimidyl ester (CFSE, Invitrogen) in PBS with 0.1% bovine serum albumin for 10 minutes at 37°C, followed by immediate quenching with cold culture medium. After 3 days, *in vitro* stimulated PBMCs were stained with anti-CD3 or anti-CD4 and tested by flow cytometry. For analysis of activation markers, PBMCs were collected after 2 days of culture and stained with indicated fluorescent-labelled human monoclonal antibodies. Cells viability was tested with annexin V/propidium iodide staining (BioLegend) according to the manufacturer’s instructions using flow cytometry.

### Cytokine detection

Supernatants of 2 x 10^5^ PBMCs, seeded into 96 wells plates, were harvested after 48 hours and stored at -80°C. Cytokine concentration was assessed by 17-cytokines multiplex ELISA kit (Bio-Plex Pro Assays, Bio-Rad) according to manufacturer’s instructions.

For intracellular cytokine staining, cells were treated overnight with 5μg/ml Brefeldin A (Sigma-Aldrich), an inhibitor of Golgi transport. Cells were detached with Tryple solution (Euroclone), stained with surface markers antibodies against CD14, CD3, CD33, CD11b, HLA-DR and fixed with 2% w/v paraformaldehyde in PBS. Then, cells were permeabilized with PBS containing 1% FBS and 0.1% saponin, incubated with fluorescent-labelled anti- IL-1β, IL-6, IL-10 or arginase-1 antibodies and analysed by flow cytometry.

### Treg cell induction and functional assays

To detect the percentage of FoxP3+ T-reg cells within stimulated CD4+ cells, CD25 surface staining and FoxP3 intracellular staining were done using Foxp3/Transcription Factor Staining Buffer Set (eBiosciences). To evaluate T-reg cell functional activity, 1 x 10^5^ isolated T-reg cells were pre-incubated with GSC-derived exosomes for 24 hours, washed and seeded in two-fold scaled dilution (from 1:1 to 1:64) with 5 x 10^4^ CFSE-stained effector T cells (CD4+), in presence of 2 x 10^5^ mitomycin (50 μg/ml)-treated PBMC and anti-CD3 (1μg/ml). Effector T cell proliferation was evaluated after 4 days by CFSE assay.

### Labelling of GSC-derived exosomes

To investigate the ability of CD14+ monocytes to internalize GSC-derived exosomes, vesicles were labelled with DiD (Invitrogen), according to the manufacturer's protocol. Briefly, 1.5 x 10^10^ exosomes were resuspended in PBS and stained with 5μM DiD for 30 minutes at 37°C. DiD-labelled exosomes were incubated with 2 x 10^5^ isolated PBMCs for 5 hours and then cells were analysed by flow cytometry by gating either on CD14^+^, CD4^+^ or CD8^+^ PBMCs.

### Statistical analyses

Data were described using means ± standard deviation or median and range for continuous variables and percentages for categorical variables. Data were tested for normal distribution using the Kolmogorov-Smirnov test. The T-test or Wilcoxon test, as appropriate, was used to compare continuous variables between two groups. For monocyte intracellular cytokine assay, one-way ANOVA followed by Bonferroni post-test was performed, while for Treg cell suppression assay, repeated measurements one-way ANOVA followed by Bonferroni post-test was used. A p value less than 0.05 was considered significant. Statistics were performed by Prism (version 4.0c).

## Results

### GSC-supernatants contain exosomes

**Fig 1:**
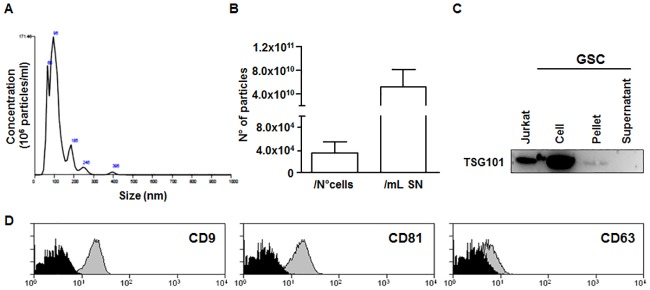
Characterization of exosomes-enriched preparation obtained from GSC. The NTA was performed on GSC-derived exosomes samples in order to quantify particle concentration normalized for the number of producing cells or millilitre of supernatants. (A) A representative graph of NTA is shown. (B) The data show the amount of exosomes produced by different GSC samples considering either the number of cells counted at the end of the 48 hours culture or the volume of cell supernatants. The data are presented as mean ± SD; n = 7. (C) Immunoblotting of the Jurkat whole cell lysate (positive control), GSC, GSC-derived ExoQuick pellet and supernatant for exosomal surface protein TSG101 (Molecular Weight, 43kDa). (D) Representative FACS histograms of CD9, CD81 and CD63 exosome-specific markers are shown.

To obtain a preparation enriched in GSC-derived exosomes, the latter were precipitated from GSC supernatants using Exoquick, a polymer-based strategy. To quantify size, distribution and particle concentration, NTA was performed (Fig 1A). The size of isolated vesicles ranged from 67.3 to 96.4nm with an average size of 86.3±10.3 nm. The concentration of GSC-derived exosomes was normalized either for the number of producing cells or for millilitre of cell supernatants (Fig 1B). Presence of exosomes was further confirmed by western blot probing for the exosomal-specific marker TSG101 (Fig 1C) and by confirming the expression of exosomal markers CD9, CD81 and CD63 by FACS analysis (Fig 1D). Similar results were obtained by analysing exosome preparations derived from astrocyte cultures (S1 Fig).

### GSC-derived exosomes inhibit T cell activation and proliferative response

**Fig 2:**
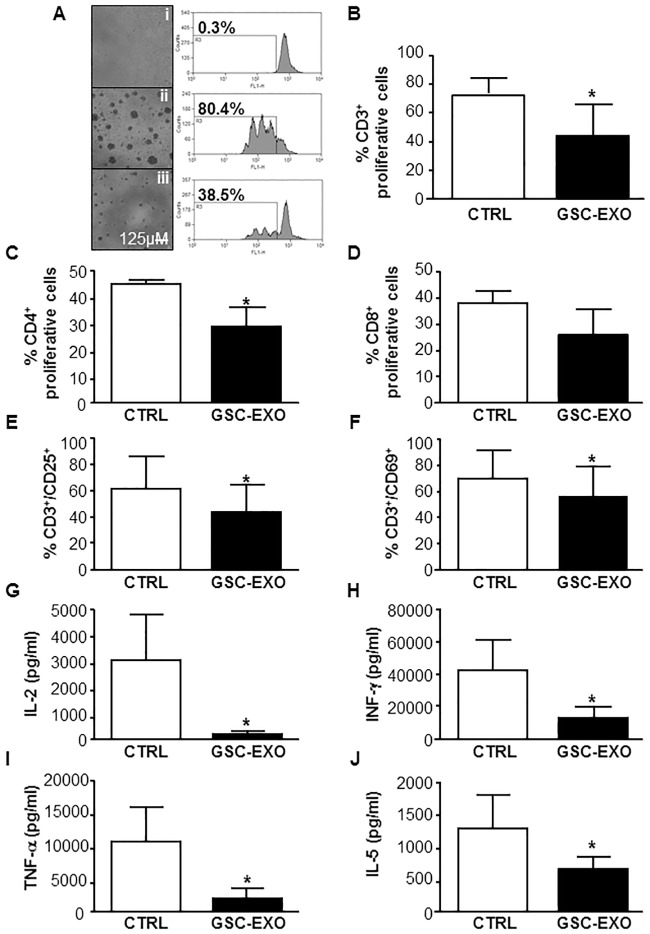
GSC-derived exosomes inhibit T-cell proliferation and expression of activation markers and modulate cytokine production of PBMCs. CFSE-labeled PBMCs isolated from healthy donors were pretreated for 24 hours without (white column, CTRL) or with GSC-derived exosomes (black column, GSC-EXO) and stimulated for 4 days with anti-CD3 and anti-CD28. (A) Representative microscope images and respective cytometry CFSE histograms, showing the fraction of proliferative CD3+ T cells, in unstimulated PBMCs (i), stimulated PBMCs (ii) and exosomes-treated stimulated PBMCs (iii). (B-D) Histograms showing, within the PBMCs, the fraction of proliferating CD3+ (B), CD4+ (C) and CD8+ (D) T cells. (E-F) CD3+ T-cell expression of CD25 and CD69 was measured by flow cytometry on day 2. (G-L) PBMC-derived supernatants were harvested after 48 hours and used for ELISA with the Bio-plex cytokine assay system. Cytokines that showed statistically significant differences with the exosome treatment are reported. Concentration of IL-2 (G), INF-γ (H), TNF-α (I) and IL-5 (J) are expressed as pg/ml. In B-L, the data are presented as mean ± SD (n = 6). *, *p*<0.05 versus control.

To determinate whether GSC-derived exosomes were acting on T cell activation and proliferation, CFSE-labelled PBMCs, isolated from healthy donors, were incubated for 24 hours with exosomes precipitated from GSC supernatants and then stimulated with plate-bound anti-CD3 and soluble anti-CD28. On day 4, to distinguish exosome effects on the proliferation of distinct T cell subsets, cultured PBMCs were stained with specific surface markers. We found that GSC-derived exosomes significantly inhibited CD3+ T cells proliferation (Fig 2A and 2B), impairing CD4+ T cells proliferation (Fig 2C) rather than CD8+ T cells proliferation (Fig 2D). In order to understand if the decreased proliferation was due to an increase in the rate of apoptotic or necrotic cells, we evaluated cell vitality with annexin V/propidium iodide test. The results indicated that the pre-incubation with GSC-derived exosomes did not influence cell viability (S2 Fig, panel A and B). Moreover, we observed that GSC-derived exosomes were able to decrease the expression of both the alpha chain of the IL-2 receptor (CD25) and the CD69 activation marker on stimulated CD3+ T cells (Fig 2E and 2F, respectively). No significant inhibition of T cell proliferation or expression of CD25 and CD69 was detected upon PBMCs incubation with exosomes isolated from supernatants of human astrocytes (S3 Fig).

To evaluate whether GSC-derived exosomes could influence the production of proinflammatory cytokines, PBMCs were stimulated in presence or absence of exosomes and after 48 hours the supernatant was harvested for cytokine profile detection. The overall results are reported in Table 1. Production of Th1 cytokines IL-2, INF-γ, and TNF-α was significantly decreased when PBMCs were stimulated in the presence of GSC-derived exosome as well as IL-5 (Fig 2G to 2J, respectively), while the Th2 cytokine IL-6 was upregulated (Table 1).

**Table 1:** Cytokine production by stimulated PBMCs treated with GSC-derived exosomes.

CYTOKINE	UNSTIM	STIM	STIM GSC-EXO
**IL-1β**	1.9±1.1	754.5±12.92 (*)	723.1±119.3 (*)
**IL-2**	23.5±25.9	2935.62±17073.4 (*)	189.9±107.4 (*, §)
**IL-4**	13.3±5.7	58.8±29.9 (*)	43.1±22.5 (*)
**IL-5**	1.9±1.2	1260±509.9 (*)	654.8±179.1 (*, §)
**IL-6**	759.5±138.7	4778.1±1742.8 (*)	8943.3±1501 (*, §)
**IL-7**	291.9±156.3	324.3±192.9	356.4±175.5
**IL-8**	6280.7±2028.6	9463.6±2165.4	8944±1714.6
**IL-10**	417.1±29.2	8481.7±3769 (*)	5278.1±1655.8 (*)
**IL-12**	32.5±20.6	209.3±275.8	75.6±41.5
**IL-13**	26.2±6.3	670.5±186.5 (*)	605.9±160 (*)
**IL-17**	281.2±359.3	3503.12±973.1 (*)	2948.3±1693.4 (*)
**G-CSF**	186.7±69.8	416.7±102 (*)	450.5±80.4 (*)
**GM-CSF**	97.9±43.1	674.2±336.8 (*)	517.1±32.8 (*)
**INF-γ**	243.7±213.7	41915.9±17671 (*)	12670.6±6711.2 (*, §)
**MCP-1**	4649.3±1041.3	4825.1±684.6	5390.5±406.1
**MIP-1β**	1335.2±335	17731.5±2066.1 (*)	21641.6±5246.7 (*)
**TNF-α**	29.2±15.9	10453.6±4904.6 (*)	1951.5±1517.6 (*, §)

PBMCs isolated from healthy donors were stimulated with anti-CD3 and anti-CD28 in absence or presence of GSC-derived exosomes. Cytokine production was measured on cell supernatants by ELISA Bio-plex cytokine assay system. Cytokine concentrations are expressed as pg/ml and all data are presented as mean ± SD of six experiments;

*, *p*<0.05 versus unstimulated control;

^§^, *p*<0.05 versus stimulated control.

These data suggest that one mechanism, by which cancer-initiating cells suppress T-cell proliferative and proinflammatory responses, is throughout the release of specific inhibitory exosomes.

### GSC-derived exosomes require intact PBMC population to inhibit T cell response

**Fig 3:**
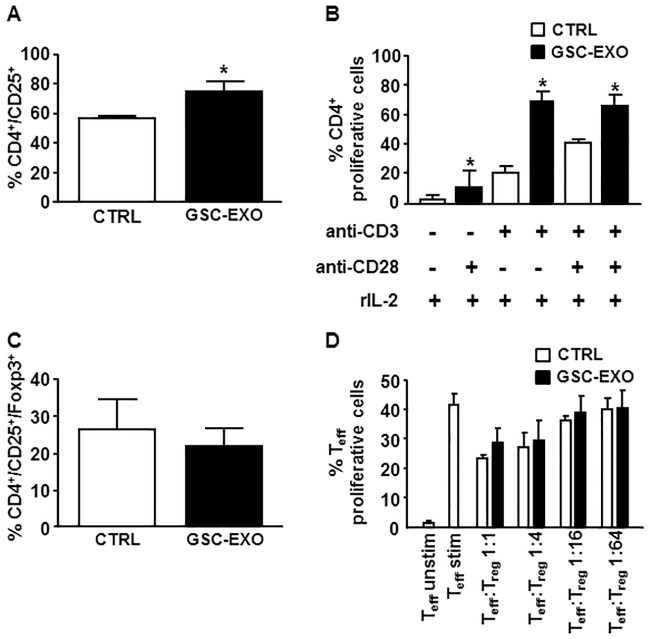
GSC-derived exosomes stimulate CD25 expression and proliferation of isolated CD4+ T cells but do not affect differentiation and suppressive activity of Treg cells. CD4+ T cells, isolated from PBMCs by negative selection, were stimulated with anti-CD3, anti-CD28 and IL-2 in the absence (white column, CTRL) or presence (black column, GSC-EXO) of GSC-derived exosomes. Expression of CD25 (A), percentage of proliferative CFSE-labelled cells in the presence of indicated stimuli (B) and frequency of CD4+/CD25+/FoxP3+ (C) was determined by flow cytometry on day 4. Columns, mean (n = 6); bars, SD; *, significantly different from the control; *p*<0.05. (D) CFSE-labelled purified CD4+ T cells, stimulated with anti-CD3, were co-cultured for 4 days with mitomycin-treated PBMCs and CD4+/CD25+/CD127^dim^ T-reg cells pre-incubated without (white column, CTRL) or with (black column, GSC-EXO) GSC-derived exosomes. Percentage of proliferative CFSE-labelled CD4+ T cells was measured by flow cytometry. Columns, mean (n = 4); bars, SD.

We next studied whether the anti-proliferative effect of GSC-derived exosomes persisted on isolated CD4+ T cells; thus, purified CD4+ T cells were pre-treated with exosomes and then activated with plate-bound anti-CD3, soluble anti-CD28 and IL-2. To our surprise, in this experimental setting the percentage of CD4+, expressing the CD25 activation marker significantly increased (Fig 3A, from 56.4±1.4% to 74.5±6.6%, p = 0.01). Moreover, co-incubation of purified CFSE-labelled CD4+ T cells with GSC-derived exosomes significantly enhanced their proliferation, indicating a stimulatory effect of GSC-derived exosomes on isolated CD4+ T cells (Fig 3B, from 41.8±6.4% without exosomes to 67.1±15.7% with exosomes, p = 0.03). Of note, the proliferation rate observed in anti-CD3 and anti-CD28 exosome-treated CD4+ T cells was also reached in the absence of the CD28 co-stimulatory signal (Fig 3B, 67.1±15.7% vs. 71±14.5%, respectively), suggesting that exosomes may act as co-stimulus for CD4+ cell activation.

We hypothesized that the suppressive effect of GSC-derived exosomes observed on T cells within the whole PBMC population could be due to an induction of T-reg cells. Actually, incubation with exosomes did not expand the percentage of CD4+/CD25+/FoxP3+ cells within stimulated PBMCs (Fig 3C). Next, to test whether the ability of T-reg cells to suppress immune response was affected by exposure to GSC-derived exosome, CD4+CD25+CD127^dim/-^ T-reg cells were purified from PBMCs and pre-treated for 24 hours with GSC-derived exosomes, before incubation with CFSE-labelled CD4+ cells, at a ratio ranging from 1:1 to 1:64, and mitomycin-treated PBMCs. GSC-derived exosomes did not alter the suppressive activity of Treg cells (Fig 3D), therefore suggesting that tumor-derived exosomes exert a suppression of T cell-effector function by acting on PBMC cells other than Treg.

### Monocytes, responding to GSC-derived exosomes, induce CD3+T-cell suppression and change their own cytokine profile

**Fig 4:**
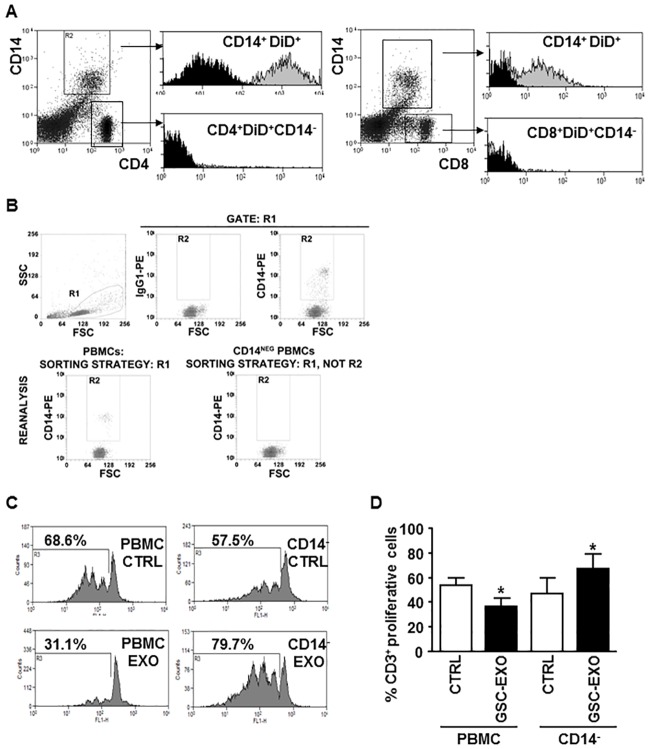
GSC-derived exosomes are internalized by monocytes and stimulate proliferation of CD14 negatively-sorted PBMCs. Fluorescent exosomes (A) were incubated with PBMCs for 5 hours and the uptake by CD14+ monocytes, CD4+ or CD8+ T cells was measured by flow cytometric analysis in the bulk population. In representative cytometry histograms, the isotype control is in black and in grey are PBMCs cells incubated with labelled-exosomes and gated on CD14+ monocytes (left, top), on CD4+ (left, bottom) or on CD8+ T cells (right, bottom). (B) Gating and sorting strategies of PBMCs and CD14-depleted PBMC. (Top, left) physical parameters, i.e. forward scatter (FSC) and side scatter (SSC), were used to select PBMCs (gate R1). Monocytes were recognized by evaluating, in PBMCs, the expression of CD14 (gate R2, top, right panel). A PE-isotype matched antibody was used to define R2 (top, central panel). PBMCs-depleted cells were identified as cells included in R1 but not in R2. (C) Representative dot-plots showing the reanalysis of the FACS-sorted PBMCs (left panel) and CD14-depleted PBMCs (right panel). As expected, CD14-positive cells were present in the sorted PBMCs- but not in the CD14-depleted- samples. (D) Proliferation of both unfractioned PBMCs and PBMCs depleted of the CD14+ population (CD14-) was measured, after CFSE labelling assay, by flow cytometry. Cells were pre-incubated without (white column, CTRL) or with (black column, GSC-EXO) GSC-derived exosomes. Columns, mean (n = 6); bars, SD; *, significantly different from the control; P<0.05. Representative cytometry CFSE histograms of PBMCs (C-E) and CD14- cells (D-F) are shown with the percentage of proliferative cells indicated.

To elucidate the opposite effect that GSC-derived exosomes exert on total PBMCs and on fractionated CD4+ T cells, we hypothesized that exosomes could act directly on monocytes that, in turn, inhibit T cell function. To test if exosomes are internalized by monocytes, exosomes were stained with the DiD dye and incubated with PBMCs. CD14+ monocytes, but not CD4+ or CD8+ T cells effectively internalized the DiD-labelled exosomes as observed by flow cytometry (Fig 4A). Then, we examined the outcome of GSC-derived exosome on CD3+ T-cell proliferation in a PBMCs culture system depleted or not of CD14+ monocytes. To remove CD14+ monocyte, PBMCs were stained with anti-CD14 fluorescent antibody and sorted by a FACSAria II cell sorter, as described in the methods section. 99.0±0.7% of CD14 depletion was achieved (Fig 4B). CD3+ T-cell proliferation was significantly inhibited upon GSC-derived exosome treatment (from 51.1±17.1% to 37.3±17.9%; p = 0.046), but increased if monocytes were removed (from 47.5±13% to 67.7±12.3%; p = 0.015) (Fig 4C and 4D).

All these data indicate that CD14+ monocytes play a role in exosome-mediated immunosuppression.

**Fig 5:**
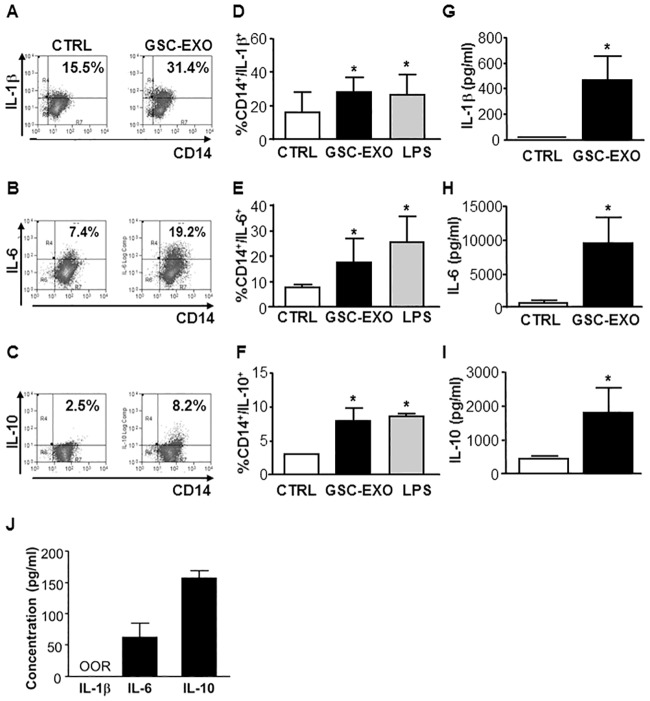
GSC-derived exosomes stimulate IL-1β, IL-6 and IL-10 production in unstimulated CD14+ monocytes within PBMC population. Unstimulated PBMCs were incubated in the absence (white column, CTRL) or presence (black column, GSC-EXO) of GSC-derived exosomes. Incubation with LPS (square column) was used as monocyte stimulation positive control. Cells were surface stained with anti-CD14 and then stained to detect an intracellular level of IL-1β, IL-6 and IL-10 by flow cytometry. (A-C) Representative FACS plot of the intracellular staining is shown by the indicated percentage of CD14+/IL-1β+, CD14+/IL-6+ and CD14+/IL-10+ positive cells, respectively. (D-F) The mean of the experiments is shown (n = 6); bars, SD; *, significantly different from the control; P<0.05. Supernatants of unsorted unstimulated PBMCs incubated in the absence (white column) or presence of GSC-derived exosomes were harvested after 48 hours and used for a cytokine assay with the Bio-plex cytokine assay system. (G-I). As a control, cytokine concentration was also tested on a medium added with isolated exosomes (J). Concentration of IL-1β, IL-6 and IL-10, respectively, are expressed as pg/ml. Columns, mean (n = 6); bars; SD; *, significantly different from the control; P<0.05.

To dissect the effect of GSC-derived exosomes on either CD14+ monocyte or CD3+ T lymphocyte cytokine response, we performed intracellular staining on exosome-treated PBMCs. GSC-derived exosomes *per se* increased the production of IL-1β, IL-6 and IL-10 by CD14+ monocyte cells to a level comparable with the monocyte activator signal triggered by LPS (Fig 5, panels A-F), while they did not affect the cytokine production by CD3+ T cells (S4 Fig). Cytokine production was also measured using multiplex ELISA kit in the supernatants of PBMCs treated with GSC-derived exosomes, and we observed a significant increase in IL-1β, IL-6 and IL-10 concentration (Fig 5, panels G-I). To ensure that these cytokines were not previously present in our exosome preparations, we tested the cytokine concentration on medium added with isolated exosomes. The results indicated that in GSC-derived exosome preparations IL-1β was absent, while IL-6 and IL-10 were present only in trace amounts (Fig 5, panel J).

**Fig 6:**
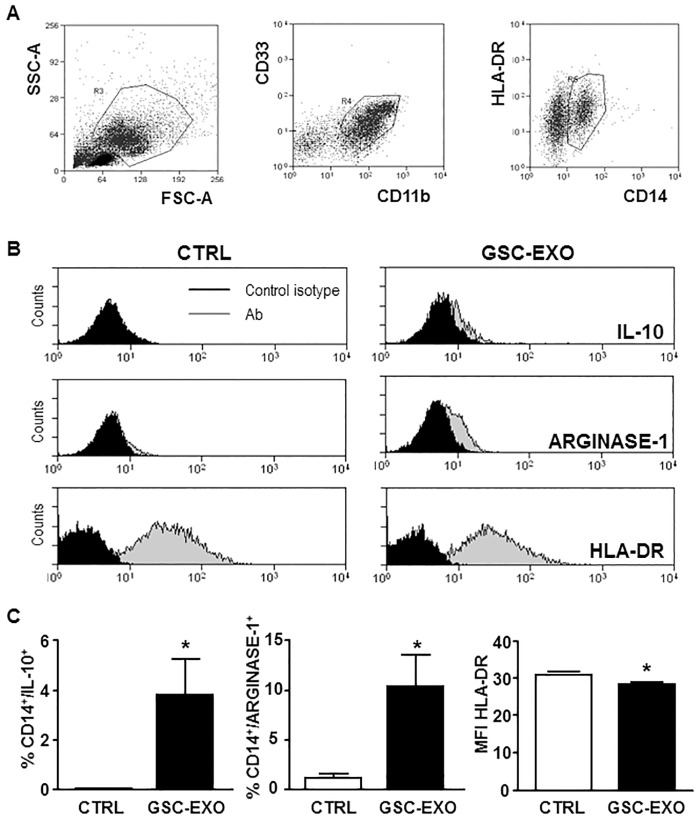
Within PBMCs population, GSC-derived exosomes promote an immunosuppressive phenotype in monocytes and stimulate the production of arginase-1 and IL-10 by Mo-MDSCs. Unstimulated PBMCs were incubated in absence (CTRL) or presence (GSC-EXO) of GSC-derived exosomes. Cells were surface stained with anti-CD14, anti CD33, anti CD11b and HLA-DR and then stained to detect intracellular level of IL-10 and arginase-1 by flow cytometry. (A) Gating strategy: physical parameters, i.e. forward scatter (FSC) and side scatter (SSC), were used to select monocytes (gate R3, left panel). Monocytes were recognized evaluating the expression of CD11b/CD33 (gate R4, middle panel) and CD14/HLA-DR (gate R5, right panel). (B) Representative FACS histograms of the intracellular staining of IL-10 and arginase-1 and of HLA-DR staining of CD14+/CD11b/CD33+ cells are shown. (C) The percentage of cells expressing IL-10 and arginase-1 and the MFI ratio of HLA-DR expression are shown (n = 6); bars, SD;*, significantly different from the control; P<0.05.

To characterize the phenotype of these suppressive monocytes within the PBMC population, cells treated for 24-hours with GSC-derived exosomes were stained to assess CD33, CD11b, CD14, HLA-DR, IL-10 and arginase-1 expression (Fig 6). We found that GSC-derived exosomes stimulated, in a population of monocytes characterized by the expression of CD33+, CD11b+, CD14+ arginase-1, the production of IL-10 (ranging from 0.03±0.01% without exosomes, to 3.8±1.4%, with exosomes; p<0.022) and the downregulation of HLA-DR (MFI from 31.2±0.3, without exosomes, to 28.5±0.5, with exosomes; p = 0.012). This immunophenotype was compatible with that of Mo-MDSC [29]. Furthermore, GSC-derived exosomes stimulated the production of Arginase-1 (from 1.1±0.3%, without exosomes, to 10.4±0.3%, with exosomes; p<0.021) an intracellular enzyme known to be a Mo-MDSC key effector in T-cell suppression [30].

### Exosomes derived from the plasma of GBM patients retain an immunosuppressive function

**Fig 7:**
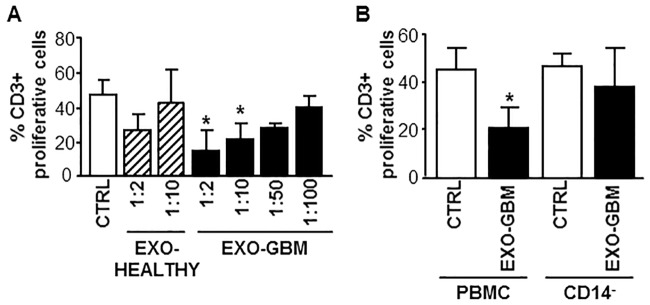
Exosomes isolated from plasma of high grade glioma patients inhibit CD3+T cells proliferation through an effect mediated by CD14+monocyte. (A) PBMCs were stimulated with anti-CD3 and anti-CD28 in the absence (white column, CTRL) or presence (black column, GBM-EXO) of different dilutions of exosomes isolated from plasma of high grade glioma patients (GBM). Healthy donor plasma-derived exosomes were used as control (striped column, EXO-HEALTHY). (B) PBMCs or CD14 negatively-sorted PBMCs (CD14-) were stimulated with anti-CD3 and anti-CD28 in the absence (white column, CTRL) or presence (black column, GBM-EXO) of exosomes isolated from plasma of GBM, 1:10 dilution. Proliferation of CD3+ was measured by CFSE assay on day 4. Columns, mean (n = 6); bars, SD;*, significantly different from the control; P<0.05.

To determinate whether circulating exosomes from GBM patients were acting on immune cells as well as the GSC-derived ones, we isolated exosomes from the plasma of ten GBM patients and we examined their effect on CD3+T cell proliferative response at different dilution (1:2, 1:10, 1:50 and 1:100). Exosomes derived from the plasma of healthy donors were used as control. GBM plasma-derived exosomes decreased the proliferation of stimulated CD3+T cells in a dose-response manner and mediated a significantly higher immune suppression than exosome-derived from the plasma of healthy donors used at the same working dilution (Fig 7A). Removal of CD14+ cells from PBMCs impaired the inhibitor effect of GBM plasma-derived exosomes (Fig 7B), as previously observed in the experiment with GSC-derived exosomes.

These data not only demonstrate that tumor-derived exosomes play a relevant clinical role in GBM patient immune response, but also ascribe to monocytes a pivotal role in orchestrating the exosome-mediated immunosuppression, at least in our experimental settings.

### GSC-derived exosomes purified by ultracentrifugation confirm their immunosuppressive mechanism of action

**Fig 8:**
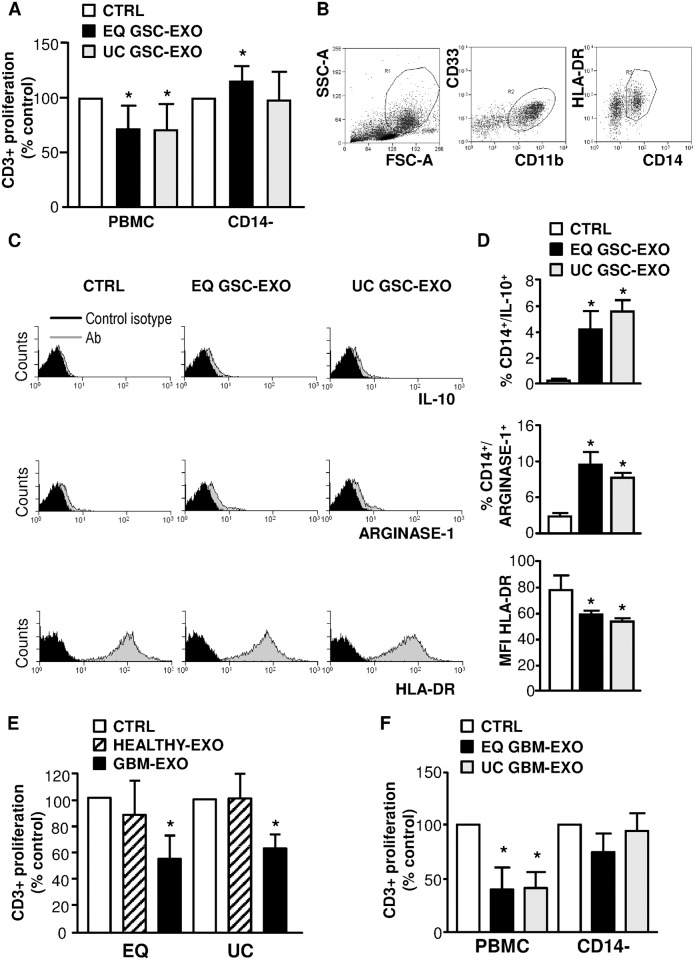
Ultracentrifugated (UC) GSC-derived exosomes promote an immunosuppressive phenotype in monocytes similarly to ExoQuick (EQ) purified GSC- or GBM-derived exosomes. (A) CFSE-labelled PBMCs isolated from healthy donors (left) or CD14 negatively-sorted PBMCs (CD14-) (right) were pre-treated for 24 hours without (white column, CTRL) or with EQ or UC isolated GSC-derived exosomes (black column, EQ GSC-EXO; grey column, UC GSC-EXO, respectively) and stimulated for 4 days with anti-CD3 and anti-CD28. Histograms show a significant difference in the percentage of proliferating CD3+ (n = 4); bars, SD;*; from the control; P<0.05. (B-D) Induction of a Mo-MDSC phenotype on monocytes. Unstimulated PBMCs were incubated in the absence (CTRL) or presence of GSC-derived exosomes purified by EQ (black column, EQ EXO-GSC) or UC(grey column, UC EXO-GSC). Cells were surface stained with (B) anti-CD14, anti CD33, anti CD11b and (C) HLA-DR and then stained to detect intracellular level of IL-10 and arginase-1 by flow cytometry. (B) Gating strategy: physical parameters, i.e. forward scatter (FSC) and side scatter (SSC), were used to select monocytes (gate R1, left panel). Monocytes were recognized by evaluating the expression of CD11b/CD33 (gate R2, middle panel) and CD14/HLA-DR (gate R3, right panel). (C) Representative FACS histograms of the intracellular staining of IL-10 and arginase-1 and of HLA-DR staining of CD14+/CD11b/CD33+ cells are shown. (D) The percentage of cells expressing IL-10 and arginase-1 and the MFI ratio of HLA-DR expression are shown (n = 3); bars, SD;*, significantly different from the control; *, P<0.05; **,P<0.01. (E) PBMCs were stimulated with anti-CD3 and anti-CD28 in the absence (white column, CTRL) or presence (black column, GBM-EXO) of exosomes isolated from plasma of glioblastoma patients (GBM) by either EQ or UC and used at 1:10 dilution. Healthy donor plasma-derived exosomes were used as a control (striped column, EXO-HEALTHY). Proliferation of CD3+ was measured by CFSE assay at day 4. Proliferation in the presence of exosomes was normalized to CD3+ cell proliferation in the absence of exosomes (CTRL set to 100%). (F) Proliferation of both unfractioned PBMCs and PBMCs depleted of the CD14+ population (CD14-) was measured, after CFSE labelling assay, by flow cytometry. Cells were pre-incubated without (white column, CTRL) or with EQGBM-derived exosomes (black column, EQ GBM-EXO) or UC GBM-derived exosomes (grey column, UC GBM-EXO). Columns, mean (n = 4); bars, SD; *, significantly different from the control; P<0.05.

To exclude that the immunosuppressive function of exosome preparations enriched by ExoQuick (EQ) was strictly related to the isolation method, GSC-derived exosomes were obtained by ultracentifugation (UC), a well-established exosome isolation method [31]. Upon purification, size distribution and particle concentration revealed that samples were similar in size, but the yield was lower in the UC than in the EQ procedure (S5 Fig), in accordance to what observed in other comparative reports [32,33]. We next examined the effects of GSC-derived exosomes, isolated by both UC and EQ on CD3+ T cell proliferation in PBMC culture, either unfractionated or upon removal of the CD14+ population (Fig 8A). CD3+ T-cell proliferation was significantly inhibited upon treatment with both UC GSC-derived exosomes (from 100% to 71±24.2%;p = 0.019) and with EQ GSC-derived ones (from 100% to 71.9±21.8%; p = 0.014). Conversely, CD3+ T-cell proliferation was not affected by UC GSC-derived exosomes when monocytes were removed (from 100% to 98.8±26.1%; p = 0.905). In the absence of CD14+ monocytes, EQ GSC-derived exosomes were instead able to induce an increase in the CD3+ T-cell proliferation, as previously observed in Fig 4, suggesting the existence of some heterogeneity in the exosome preparation obtained by the two different enrichment procedures.

To verify the ability of GSC-derived exosomes precipitated by UC to induce in the CD14+ population an immunophenotype compatible with that of Mo-MDSC, UC-purified or EQ-purified exosomes were used to treat the PBMC population for 24 hours as done in Fig 6. The characterization of the phenotype of monocytes within the PBMC population was performed by staining cells to assess the expression of CD33, CD11b, CD14, HLA-DR, IL-10 and arginase-1 (Fig 8B–8D). Again, a population of monocytes characterized by the expression of CD33+, CD11b+, CD14+ IL-10, Arginase-1 and downregulation of HLA-DR, was detected in the cultures treated with both types of exosomes. Lastly, to determinate whether circulating exosomes from GBM patients purified by UC were acting on immune cells as the ones purified by EQ, we tested their effect on the CD3+ T cell proliferative response. Exosomes derived from the plasma of healthy donors were used as a control. GBM plasma-derived exosomes, regardless of the purification procedure used in the experiments (Fig 8E), decreased the proliferation of stimulated CD3+ T cells and mediated a significantly higher immune suppression than exosomes derived from the plasma of healthy donors used at the same working dilution (from 100% to 40.8±16.4% for UC GBM-derived exosomes, p = 0.025; from 100% to 39.6±20.6% for EQ GBM-derived exosomes, p = 0.036). Again, the removal of the CD14+ monocytic population abrogated the inhibitory effect (Fig 8F) of both UC GBM-derived exosomes (from 100% to 94.5±17.8, p = 0.648) and EQ GBM-derived exosomes (from 100 to 74.5±18.4, p = 0.139).

In conclusion, GSC-derived and GBM plasma-derived exosomes are immunosuppressive acting on monocytes, regardless of the type of isolation procedure adopted.

## Discussion

In this report, we elucidated the immunosuppression pathway mediated by exosomes produced by GSC. We demonstrated that monocytes, responding to GSC-derived exosomes, mediate the CD3+ T cell suppression, through the de-differentiation into Mo-MDSC. Thus, the paper highlights a clear role of GSC-derived exosomes in glioma cell evasion from the immune surveillance.

It has already been reported that GSC, by either direct cell-to-cell contact and/or secreted immunosuppressive cytokines or modulating factors, would induce T-cell apoptosis and T-reg cells, hence inhibiting T-cell proliferation and activation [12]. Here, we pointed out that the documented immunosuppression properties of GCS are also mediated by the release of exosomes, thus explaining the systemic immunosuppression observed in GBM patients. In fact, exosomes are usually transported through biological fluids such as plasma or serum and could act on site different from that of the cells of origin [15].

Compared with previous studies, we evaluated the immunosuppressive effect of GSC-derived exosomes on CD4+ and CD8+ T cells, monocytes, Treg cells within PBMCs, rather than on purified immune cell subpopulations. In accordance with Hellwinkel el al. [23], we reported that GSC-derived exosomes downregulate the activation of PBMCs in terms of proliferation and cytokines production. We demonstrated that the proliferation of CD4+ T within PBMCs treated with GSC-derived exosomes was inhibited, whereas there were no significant effects on CD8+ T cells, supporting the results obtained in [34], in which glioma-derived exosomes failed to alter the activation and INF-γ production by CD8+ T cells.

We found that GSC-derived exosomes were able to promote proliferation of activated purified CD4+T cells and to stimulate the expression of the CD25 activation marker, but unexpectedly, CD4+CD25highFOXP3+ T-reg did not represent these proliferating CD4+ T cells. Indeed, the data reported by Wieckowski et al. [35] showed that MVs purified from melanoma and neck squamous carcinoma cell lines induce the *in vitro* expansion of CD4+CD25+FOXP3+ T-reg cells and enhance their suppressor activity. This discrepancy could be due to a different tumor origin and to the type of EVs analysed, MVs in the quoted paper and the exosomes in our work. MVs bud directly from the plasma membrane, which blebs and packages the cellular components in defined structures that are then released into the extracellular environment upon cell activation, whereas exosomes derive from the inward budding of the late endosome, or multivesicular bodies, and are a more homogeneous population [17]. Therefore, although the overall function of tumor-derived EVs seems to support tumor escape, our results suggest that the biological effect on fractionated immune cells could vary accordingly to the type of vesicles analysed [36].

Proliferation of CD4+ even in absence of the either CD3 or CD28 co-stimulatory signal suggests that vesicles express co-stimulatory molecules on their surface membrane. Indeed, it was recently shown that T cells do not internalize tumor-derived exosomes, consequently exosomes deliver signals to receptors present on the cell surface, which ultimately results in alterations of the mRNA profile [37]. However, it has been reported that glioma cell lines-derived exosomes lack antigen-presentation and the surface co-modulatory machinery CD80 and CD86 [34] able to cross-react with CD28. It is probable that the presence of other costimulatory molecules such as CD275 (Inducible T-cell co-stimulator ligand, ICOSL) or CD252 (OX40 ligand), which are expressed on glioma cells [38,39], could account for the result that we have observed. A new set of experiments would be required to investigate the implications of this finding, even if this lies outside of the objectives of this work.

On the other hand, we demonstrated that GSC-derived exosomes were efficiently internalized by CD14+ monocytes and were able to stimulate the production of IL-1β, IL-6 and IL-10 cytokines. It is widely recognized that abnormalities in cytokine expression are implicated in gliomagenesis [40] consequently, it is possible to hypothesize that GSC, through the release of exosomes, are able to educate immune cells to produce cytokines for their own survival. Specifically, IL-6 is heavily involved in glioma development through the promotion of angiogenesis, cell proliferation and resistance to apoptosis and radiation [41–43]. Expression levels of IL-6 have also been inversely associated with glioma patient’s survival because of the role played in GBM growth [44]. Beside, IL-10 mRNA correlate with tumor grade [45], increases cell proliferation and motility of glioma cells [45,46] and is mainly concentrated in microglia and macrophages which contribute to tumor progression though the inhibition of the patient’s immune response [47].

However, the key finding of our work is that the inhibition of CD4+ cells effector function observed in un-fractioned PBMCs is due not to the direct delivery of exosomes to T cells, but to the activation of monocytes, which acquire a more immature immunophenotype skewed towards the expansion of Mo-MDSC. In fact, we showed that exosomes are able to downregulate T cells’ response only in the presence of monocytes. We are aware that the technique used to purify exosomes could be crucial in obtaining a homogenous population [48–50]. Although the comparison between different techniques was beyond the purpose of this work, to prove that the effects of tumor-derived exosomes on the immune system were due to exosome content and not to non-vesicular contaminants, we have performed key experiments not only with EQ purified vesicles, but also with exosomes purified by UC, a well-established method used to isolate a homogenous size exosome population. We achieved similar results with both types of preparations, thus further strengthening the immunosuppressive function of tumor-derived exosomes.

It has previously been demonstrated that GSC–conditioned medium inhibits monocytes phagocytosis, induces the secretion of IL-10 [13] and impairs T-cell function toward an alteration of the cytokines profile of monocytes [9]. Recently, it has also been reported that GBM-derived EVs skew the differentiation of peripheral blood-derived monocytes to alternatively activated/M2-type macrophages [24]. In our work we provide evidence that GSC-derived exosomes are involved in the conversion of monocytes to arginase-1- and IL-10-producing Mo-MDSC cells that contribute to T cells immunosuppression without the necessity of direct contact between monocytes and glioma cells. EVs from GBM cells were reported to contain cell-transforming proteins, mRNAs and specific types of small noncoding miRNAs and are able to modify recipient cells of tumor or endothelial origin [24,51]. Moreover, it was seen that monocytes/macrophages within the brain avidly took up GBM-derived macrovesicles, leading to increased proliferation and a modification in their cytokine profile toward immune suppression partially associated with increased miRNA levels and decreased target mRNAs [52]. Surely, further work in the area of characterization of GSC-derived exosome content is needed.

## Conclusion

Our results suggest that GSC-derived- as well as exosomes isolated from the peripheral blood of GBM patients are able to induce peripheral T cell immunosuppression by acting on monocytes and by skewing them toward a Mo-MDSC tumor-supportive phenotype. As tumor-derived exosomes represent a mechanism for intercellular communication containing mediators of tumor progression, they could be envisaged as tools to reprogram antitumor immunity and to restore effector functions in immunosuppressed cells. For this purpose it would be necessary to gain more insight into the biology of GSC-derived exosomes which may lead to novel therapies [53].

## Supporting Information

S1 Fig Characterization of exosomes-enriched preparation obtained from astrocytes. The NTA was performed on astrocytes-derived exosome samples in order to quantify particle concentration normalized for number of producing cells or milliliter of supernatants. (A) The NTA data are presented as mean ± SD; n = 3. (B) Representative FACS histograms of CD9, CD81 and CD63 exosome-specific markers are shown.
(TIF)

S2 Fig GSC-derived exosomes do not influence cell viability. PBMCs from healthy donors were stimulated with anti-CD3 and anti-CD28 in absence (white column, CTRL) or presence (black column, GSC-EXO) of GSC-derived exosomes. The percentage of apoptotic (A) and necrotic (B) cells was tested by flow cytometry with annexinV/propidium iodide staining at day 3. Columns, mean (n = 6); bars, SD.
(TIF)

S3 Fig Astrocyte-derived exosomes do not affect T cells proliferation and expression of activation markers. PBMCs isolated from healthy donors were stimulated with anti-CD3 and anti-CD28 in absence (white column, CTRL) or presence (grey column, ASTROCYTE-EXO) of astrocytes-derived exosomes. Proliferation of CD3+ (A) and expression of CD25 (B) and CD69 (C) was measured by flow cytometry as described in material and methods section (columns, mean n = 3; bars, SD).
(TIF)

S4 Fig GSC-derived exosomes do not stimulate IL-1β, IL-6 and IL-10 production in unstimulated CD3 within PBMC population. Unstimulated PBMCs were incubated in absence (white column, CTRL) or presence (black column, GSC-EXO) of GSC-derived exosomes. Cells were surface stained with anti-CD3 and then stained to detect intracellular level of IL-1β, IL-6 and IL-10 by flow cytometry. (**A-C**) The mean of the percentage of C3^+^/IL-1β^+^, CD3^+^/IL-6^+^ and CD3^+^/IL-10^+^ positive cells, respectively (n = 3); bars, SD.
(TIF)

S5 Fig Characterization of GSC-derived exosomes obtained by ultracentrifuge. The NTA was performed on ultracentrifuged GSC-derived exosomes in order to quantify particle concentration normalized for number of producing cells or milliliter of supernatants. (A) A representative graph of NTA is shown. (B) The data show the amount of exosomes produced by different GSC samples considering either the number of cells counted at the end of the 48 hours culture or the final volume of cell supernatants. The NTA data are presented as mean ± SD; n = 3.
(TIF)
