# Supplementary figures and images for: Systemic T Cells Immunosuppression of Glioma Stem Cell-Derived Exosomes Is Mediated by Monocytic Myeloid-Derived Suppressor Cells
Source: PLoS One. 2017 Jan 20;12(1):e0169932. doi: 10.1371/journal.pone.0169932 (PMC5249124; doi:10.1371/journal.pone.0169932)

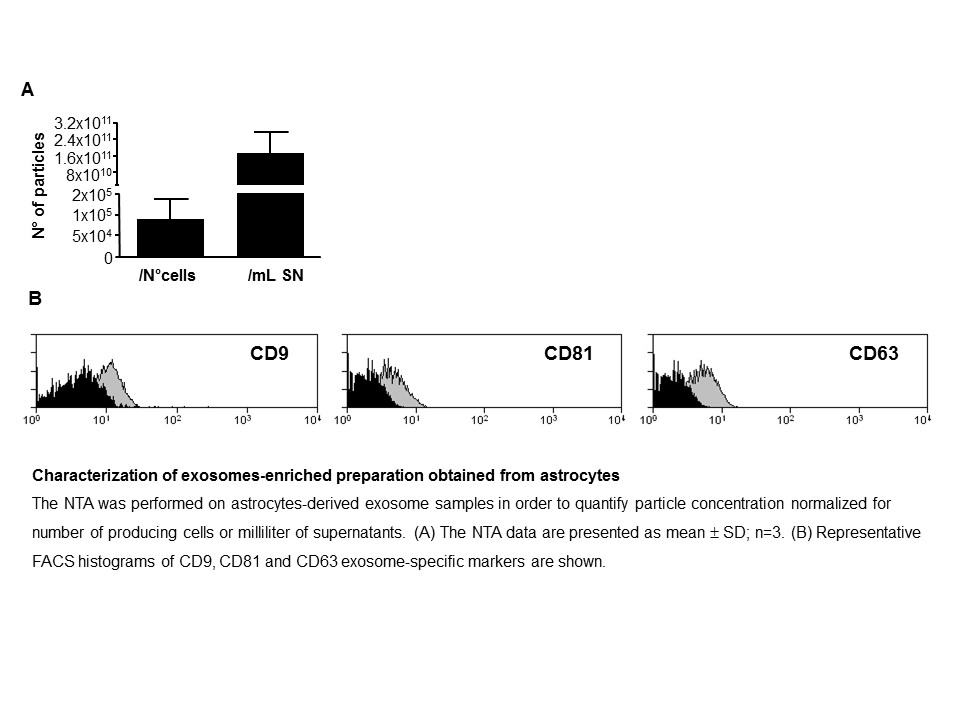

Supplement: S1 Fig — The NTA was performed on astrocytes-derived exosome samples in order to quantify particle concentration normalized for number of producing cells or milliliter of supernatants. (A) The NTA data are presented as mean ± SD; n = 3. (B) Representative FACS histograms of CD9, CD81 and CD63 exosome-specific markers are shown. (TIF) [file pone.0169932.s001.tif]

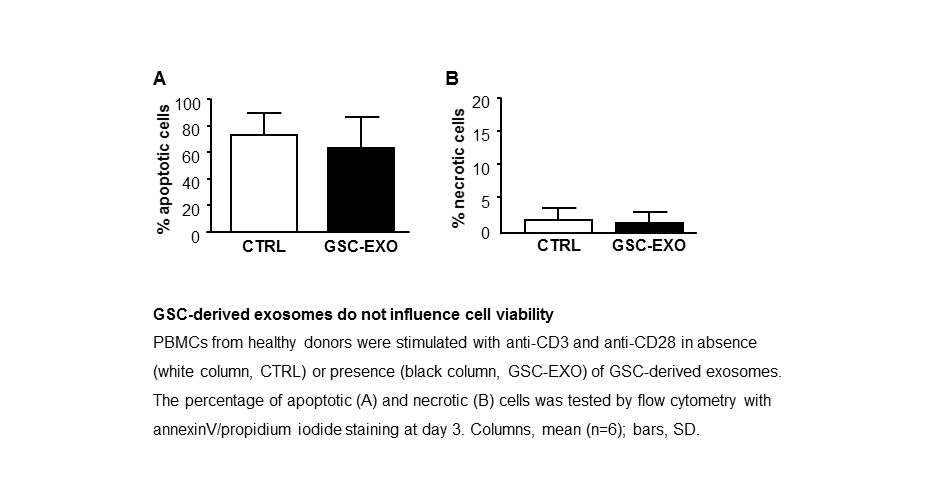

Supplement: S2 Fig — PBMCs from healthy donors were stimulated with anti-CD3 and anti-CD28 in absence (white column, CTRL) or presence (black column, GSC-EXO) of GSC-derived exosomes. The percentage of apoptotic (A) and necrotic (B) cells was tested by flow cytometry with annexinV/propidium iodide staining at day 3. Columns, mean (n = 6); bars, SD. (TIF) [file pone.0169932.s002.tif]

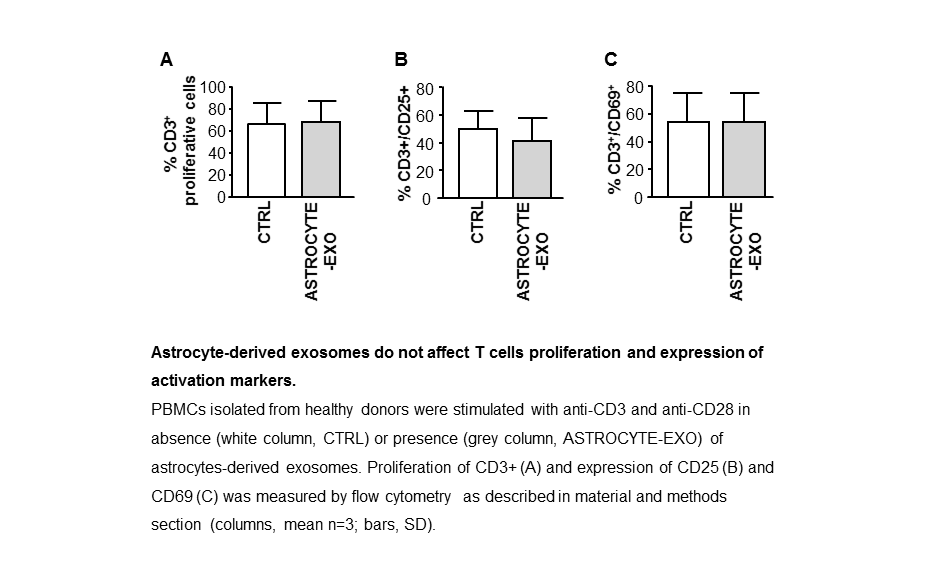

Supplement: S3 Fig — PBMCs isolated from healthy donors were stimulated with anti-CD3 and anti-CD28 in absence (white column, CTRL) or presence (grey column, ASTROCYTE-EXO) of astrocytes-derived exosomes. Proliferation of CD3+ (A) and expression of CD25 (B) and CD69 (C) was measured by flow cytometry as described in material and methods section (columns, mean n = 3; bars, SD). (TIF) [file pone.0169932.s003.tif]

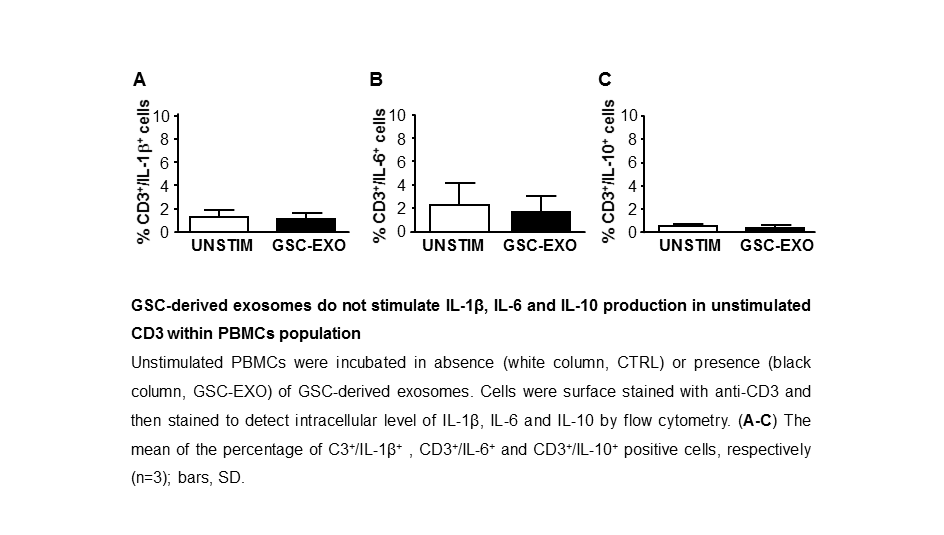

Supplement: S4 Fig — Unstimulated PBMCs were incubated in absence (white column, CTRL) or presence (black column, GSC-EXO) of GSC-derived exosomes. Cells were surface stained with anti-CD3 and then stained to detect intracellular level of IL-1β, IL-6 and IL-10 by flow cytometry. (A-C) The mean of the percentage of C3+/IL-1β+, CD3+/IL-6+ and CD3+/IL-10+ positive cells, respectively (n = 3); bars, SD. (TIF) [file pone.0169932.s004.tif]

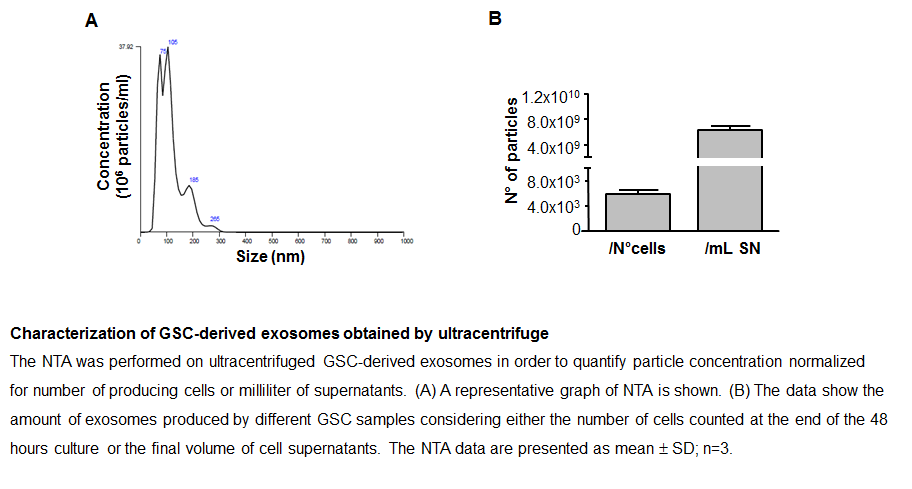

Supplement: S5 Fig — The NTA was performed on ultracentrifuged GSC-derived exosomes in order to quantify particle concentration normalized for number of producing cells or milliliter of supernatants. (A) A representative graph of NTA is shown. (B) The data show the amount of exosomes produced by different GSC samples considering either the number of cells counted at the end of the 48 hours culture or the final volume of cell supernatants. The NTA data are presented as mean ± SD; n = 3. (TIF) [file pone.0169932.s005.tif]
